# Relationship between the IL-1β serum concentration, mRNA levels and rs16944 genotype in the hyperglycemic normalization of T2D patients

**DOI:** 10.1038/s41598-020-66751-x

**Published:** 2020-06-19

**Authors:** Andrea Elena Iglesias Molli, María Fernanda Bergonzi, Mónica Paula Spalvieri, María Amelia Linari, Gustavo Daniel Frechtel, Gloria Edith Cerrone

**Affiliations:** 1 Universidad de Buenos Aires. CONICET. Instituto de Inmunología, Genética y Metabolismo (INIGEM). Laboratorio de genética de la Diabetes y del Metabolismo, Ciudad Autónoma de Buenos Aires, Argentina; 2 Universidad de Buenos Aires. Hospital de Clínicas “José de San Martín”, Ciudad Autónoma Buenos Aires, Argentina; 3 Unión Obrera Metalúrgica (UOM). Sección de Endocrinología y Nutrición, Vicente López, Argentina; 4 Universidad de Buenos Aires. Facultad de Farmacia y Bioquímica. Departamento de Microbiología, Inmunología, Biotecnología y Genética. Cátedra de Genética, Ciudad Autónoma de Buenos Aires, Argentina

**Keywords:** Genetics, Molecular biology, Medical research, Molecular medicine

## Abstract

To evaluate Interleukin 1-beta (IL-1β) serum and mononuclear leucocyte mRNA levels, also rs16944 (−511C/T) genotype, in relation to hyperglycemic normalization in Type 2 diabetes (T2D) patients, we recruited 30 individuals recently T2D diagnosed with hyperglycemia studied at basal time and after 6 and 12 months of the normalization treatment. At basal time, the T polymorphic allele of the rs16944 was associated with lower IL-1β mRNA expression (p = 0.006); and higher glucose level was positive correlated to IL-1β protein levels (p = 0.015). After treatment, the individuals showed a significant decrease in glucose level (p = 0.003), but they did not express significant changes in the IL-1β serum levels. Surprisingly, we observed that the greater decreases in glucose level were associated to increased IL-1β serum levels (p = 0.040). This is the first follow-up study evaluating IL-1β mRNA expression and serum levels in hyperglycemic T2D individuals and after glycemic normalization treatment. The current results contribute to the knowledge of the relationship between inflammation and glucose metabolism in T2D.

## Introduction

Diabetes is a major public health concern which in recent decades has become a pandemic of uncontrollable proportions^1^. About 90% of the individuals with diabetes have type 2 diabetes (T2D). In Argentina the prevalence of T2D is close to 12%, similar to other Latin American countries and most of the industrialized countries^2,3^. Like many metabolic disorders, T2D is characterized by a systemic subclinical inflammation state, secondary to innate immunity hyperactivity^4^ related with an increase in the expression of pro-inflammatory cytokines. Interleukin 1-beta (IL-1β) is one of the most important pro-inflammatory molecules related to the systemic subclinical inflammation in T2D^5–9^.

The deleterious effects of IL-1β on pancreatic β-cells are significantly increased in hyperglycemic states. It has been reported that increasing concentrations of glucose cause a rise in the production and release of IL-1β in pancreatic islets^10^. Both persistent and postprandial hyperglycemia have induced the expression of the IL-1β gene in rats, as evidenced by increased levels of IL-1β protein and mRNA in peripheral blood monocytes^11,12^. Furthermore, in a Japanese population study, circulating levels of IL-1β were found to positively correlate with fasting plasma glucose (FPG) levels, with a stronger correlation at FPG levels close to 125 mg/dL^13^. It was also reported that circulating levels of IL-1β were associated with the consumption of foods with high glycemic indexes in men and women with metabolic syndrome, a pre-diabetic state^14^.

T2D is one of the most complex, prevalent and heterogeneous diseases whose etiology is known to involve multiple interactions between genetic predisposing factors and environmental triggers^15^ but still remains to be fully elucidated. Single nucleotide polymorphisms (SNPs) constitute the genetic basis of prevalent complex diseases and, in particular, functional SNPs described in the IL-1β gene have been associated with metabolic diseases^16^.

Given that the influence of micro environmental situations such as hyperglycemia on the expression levels of these pro-inflammatory molecules has not yet been explored in individuals with T2D, the principal aim of our work was to study IL-1β mRNA expression in mononuclear leucocytes and IL-1β levels in serum in T2D hyperglycemic individuals at three different time points, i.e. upon diagnosis, and after 6 and 12 months of treatment to achieve normoglycemia. We also evaluated the relationship between mRNA expression, serum levels of IL-1β, and the rs16944 SNP (-511C/T) genotype present in the promoter of the IL-1β gene.

## Materials and Methods

### Population

A prospective controlled study was conducted in individuals with T2D where the same individual was analyzed pre and post-intervention with pharmacological treatment and changes in lifestyle. Over a period of two years, 30 individuals were randomly recruited with recently diagnosed T2D and hyperglycemia characterized by glycosylated hemoglobin (HbA_1c_) levels greater than 8% (64 mmol/mol). Out of these 30 individuals, 27 completed the prospective protocol and constituted the pre-intervention group (0 months of treatment). Inclusion criteria were: unrelated adults over 18 years of age residing in urban areas of Buenos Aires. The exclusion criteria were: acute myocardial infarction, stroke, pregnancy, psychiatric disease, alcoholism, drug abuse, history or suspicion of pancreatitis, recent intake of hyperglycemic agents (<3 months), recent cancer and instability in body weight (loss > 3 kg in the last 6 months).

Each individual received personalized pharmacological treatment and underwent changes in diet and lifestyle to achieve the target metabolic control projected as HbA_1c_ under 7% (53 mmol/mol). The pharmacological treatment of choice in 19 individuals was metformin in doses between 500 and 2550 mg/day; 5 individuals received combined treatment with metformin and insulin; 1 individual received metformin and glibenclamide; 1 individual received vildagliptin; and 1 individual did not receive pharmacological treatment.

All procedures performed in studies involving human participants were in accordance with the ethical standards of the institutional research committee and with the 1964 Helsinki Declaration and its later amendments or comparable ethical standards. The study was approved by the Ethics Committees of the Hospital de Clínicas “José de San Martín” in Ciudad Autónoma de Buenos Aires, and all participants gave their written informed consent.

All individuals informed their age and gender. Anthropometric measurements (height, weight and waist circumference), and systolic and diastolic blood pressure (SBP and DBP, respectively) were determined by standardized protocols. Body mass index (BMI) was calculated as weight (kg) / [height (m)]^2^. After 12 hours of overnight fasting, venous blood samples were drawn of every individual. Two aliquots of blood anticoagulated with EDTA K_2_ were reserved for mRNA and DNA extraction, and one aliquot of serum was reserved for measurement of IL-1β serum concentrations. All biochemical determinations were done immediately (<6 hours). FPG was determined using standardized procedures in plasma obtained by the centrifugation of blood anticoagulated with sodium fluoride. Triglycerides (TG), total cholesterol (TC), high-density lipoprotein cholesterol (HDL-C) and low-density lipoprotein cholesterol (LDL-C) were determined in serum using standardized procedures. HbA_1c_ was determined by an immunoturbidimetric method in an Architect system, with calibrators and controls that correlated with the national standardization program in the USA (NGSP and IFCC). The reference values for HbA_1c_ were 4.40–6.40% (25–46 mmol/mol) for non-diabetic, and 6.00–8.00% (42–64 mmol/mol) for controlled diabetic individuals.

### Quantification of serum IL-1β levels

IL-1β concentrations were measured in serum stored at −80 °C for short periods (<1 month) by chemiluminescence (Immulite – Siemens, DPC), according to the manufacturer’s instructions with a sensitivity of 1.5 pg/mL and a measurement range of 1.5-1000 pg/mL. The reference value for serum IL-1β was <5 pg/mL.

### Relative quantification of IL-1β mRNA expression

The aliquot of blood reserved for mRNA extraction was processed immediately. Mononuclear cells were separated with Ficoll (FicollPaque Plus, GE Healthcare) and resuspended in guanidinium thioisocyanate (TRIzol, Invitrogen Corp., Carlsbad, CA) for subsequent extraction of total RNA. Under these conditions, samples were stored at −80 °C for a maximum period of 3 months. The quality of the extracted RNA was checked on 1% agarose gels. The possible remaining DNA was removed with DNAseI (Amplification Grade DNAseI, Life Technologies). RNA was reverse-transcribed with Moloney Murine Leukemia Virus Reverse Transcriptase (M-MLV RT, Invitrogen Corp., Carlsbad, CA) and DNA hexamers as random primers (Life Technologies) to obtain the first strand of the complementary DNA (cDNA). Gene expression was analyzed by quantitative real time PCR with the StepOne system (Applied Biosystems), using the following primers: IL-1β: forward 5′-ATGATGGCTTATTACAGTGGCAA-3′ and reverse 5′-GTCGGAGATTCGTAGCTGGA-3′; and GADPH: forward 5′-TGCACCACCAACTGCTTAGC-3′ and reverse 5′-GGCATGGACTGTGGTCATGAG-3′ (Applied Biosystems). PCRs were performed in a final volume of 20 µL containing 20 ng RNA, 1X SYBR Green Master Mix (SYBR Select, Applied Biosystems) and 250 nM of each primer. The PCR conditions were: 2 min at 50 °C and 10 min at 95 °C, followed by 40 cycles of 15 sec at 95 °C and 1 min at 60 °C and a melting curve was performed in every assay to evaluate the specificity of the primers with 1 cycle of 15 sec at 95 °C, 1 min at 60 °C, and 1 min at 95 °C with a temperature ramp of 0.1 °C/sec. Each sample was analyzed in duplicate, with GADPH as normalizing control gene. The pre-intervention and both post-intervention samples for each individual were amplified in the same assay, and all measurements included the determination of a no-template negative control in which the cDNA was substituted by water. The 2^−ΔΔCt^ method was employed for relative quantification of gene expression^17^. ΔCt was calculated as the difference between the Ct of the target gene (IL-1β) minus the Ct of the endogenous control gene (GADPH) for each sample. For the pre-intervention group analysis, ΔΔCt was calculated as the ΔCt of each pre-intervention sample minus the average ΔCt of all pre-intervention samples, and the 2^−ΔΔCt^ calculated represented the fold change in mRNA expression of each pre-intervention sample in comparison with the average expression. For the prospective analysis, ΔΔCt was calculated as the ΔCt of the post-intervention sample (6 and 12 months) minus the ΔCt of the pre-intervention sample (0 months), and the 2^−ΔΔCt^ calculated represented the fold change in mRNA expression after treatment. We also calculated the 2^−ΔCt^ of each sample to evaluate the IL-1β mRNA expression levels normalized by the endogenous control at each time of the study.

### Genotyping of rs16944

We genotyped the rs16944 (-511C/T), located in the promoter region of the IL-1β gene. The aliquot of blood reserved for DNA extraction was conserved at -4 °C for short periods of time (<3 months) until its use. DNA was extracted by the cetyltrimethylammonium (CTAB) method^18^ quantified with a Qubit 2.0 Fluorometer (Invitrogen Corp., Carlsbad, CA, USA). DNA samples were prepared at concentrations of 3.3 ng/μL and genotyped by KASP (Kompetitive Allele Specific PCR) performed by LGC Genomics (http://www.lgcgroup.com). KASP technology is a uniplex SNP genotyping platform developed by LGC Genomics which utilizes a competitive allele-specific PCR. The KASP assay was carried out with primers with a universal fluorescent resonance energy transfer (FRET) cassette (FAM and HEX), ROX passive reference dye, Taq polymerase, free nucleotides and MgCl_2_ in an optimized buffer solution. The primer sequences were specific to the SNP to be targeted and were designed by LGC Genomics. They consisted in two competitive allele-specific forward primers and one common reverse primer. Each forward primer incorporates an additional tail sequence that corresponds with one of two universal FRET cassettes.

### Statistical evaluation

Statistical analyses were performed using SPSS version 20.0 (IBM Corp., Armonk, NY, USA). A p value below 0.05 was considered statistically significant. We considered the logarithms of the 2^−ΔΔCt^ values for the analysis of mRNA expression and the logarithms of the pg/mL for the analysis of serum IL-1β levels. In the pre-intervention group, we used a multiple linear regression with age, gender and weight as covariates to assess the association between IL-1β mRNA expression, serum IL-1β levels and biochemical and clinical variables. To evaluate the effect of hyperglycemia on IL-1β expression, we created groups with different levels of HbA_1c_ (<10% (<86 mmol/mol); 10-11.99% (86- 107- mmol/mol); ≥12% (108 mmol/mol)) and FPG ( < 140 mg/dL; 140-199 mg/dL; ≥200 mg/dL) and compared IL-1β mRNA expression and serum IL-1β concentrations in each level by multiple linear regression, with age, gender and weight as covariates.

The Hardy-Weinberg equilibrium was assessed by Chi square test. The association between the rs16944 SNP genotype (through dominant, recessive and allelic load models) and IL-1β mRNA expression was also analyzed by multiple linear regression, with age, gender and weight as covariates. The biochemical and clinical variables and the levels of gene expression in the individuals in each stage of the study were compared by Friedman test. IL-1β mRNA expression was analyzed by comparing the logarithms of the 2^−ΔCt^ values of the pre and post-intervention stages. The variations in serum IL-1β levels and biochemical and clinical variables were calculated as the value of the post-intervention sample (6 and 12 months) minus the value of the pre-intervention sample (0 months). The fold change in IL-1β mRNA expression and variations in serum protein levels were compared with the variations in biochemical and clinical variables, with the rs16944 SNP genotype, with the type of treatment and with the dose of each antidiabetic drug, by multiple linear regression with age, gender and BMI as covariates.

### Ethical approval

All procedures performed in studies involving human participants were in accordance with the ethical standards of the institutional research committee (Ophthalmology Clinic, Department of Medicine and Science of Ageing) and with the 1964 Helsinki Declaration and its later amendments or comparable ethical standards.

### Informed consent

Informed consent was obtained from all individual participants included in the study.

## Results

### Pre-intervention group analysis

The overall population consisted of 7 women (23.33%) and 23 men (76.67%), with a mean age of 48.17 years (SD = 12.37; range: 23–69 years). Table 1 shows the biochemical and clinical profile of the pre-intervention group, which was the expected one for individuals with T2D.

**Table 1:** Biochemical and clinical characteristics of the population at each time of the study.

	Pre-intervention group (n = 30)	Prospective controlled study					
		(n = 27)					
		Time of intervention (month)			Statistical evaluation	*post-hoc* test	
		0	6	12		0–6	0–12
	m (IQR)	m (IQR)	m (IQR)	m (IQR)	P	p	p
Weigth (kg)	91.75 (81.65–113.63)	87.00 (81.50–107.00)	86.00 (78.50–103.20)	88.40 (78.20–99.50)	0.033	0.035	NS
BMI (kg m^−2^)	33.17 (30.82–38.64)	32.91 (30.12–37.92)	31.23 (29.21–37.74)	31.91 (28.88–36.93)	0.033	0.035	NS
Waist circumference (cm)	106.0 (100.0–116.5)	102.5 (99.5–114.5)	100.0 (96.5–120.0)	106.00 (100.00–117.5)	NS	NS	NS
SBP (mmHg)	133 (120–153)	135 (120–150)	120 (120–140)	120 (120–140)	NS	NS	NS
DBP (mmHg)	80 (65–90)	80 (65–90)	70 (70–80)	75 (70–84)	NS	NS	NS
HbA_1c_ (%)	9.13 (8.09–11.23)	9.03 (8.05–11.22)	6.34 (5.84–7.03)	6.13 (5.74–6.81)	<0.001	<0.001	<0.001
FPG (mg dL^−1^)	153 (132–268)	147 (131–265)	108 (98–136)	114 (102–120)	<0.001	<0.001	<0.001
TC (mg dL^−1^)	211 (181–235)	210 (180–232)	196 (171–232)	196 (162–218)	NS	NS	NS
HDL-C (mg dL^-1^)	40 (34–47)	39 (33–47)	41 (37–45)	43 (38–51)	0.026	NS	0.027
LDL-C (mg dL^-1^)	122 (105–143)	120 (104–140)	111 (94–141)	109 (94–131)	NS	NS	NS
TG (mg dL^−1^)	153 (115–143)	150 (115–247)	200 (133–259)	148 (94–239)	NS	NS	NS

BMI: body mass index; SBP: systolic blood pressure; DBP: diastolic blood pressure; HbA_1c_: glycated hemoglobin; FPG: fasting plasma glucose; TC: total cholesterol; HDL-C: high-density lipoprotein cholesterol; LDL-C: low-density lipoprotein cholesterol; TG: triglycerides; m: median; IQR: interquartile range; NS: not significant. Statistical evaluation: Friedman test.

**Figure 1:**
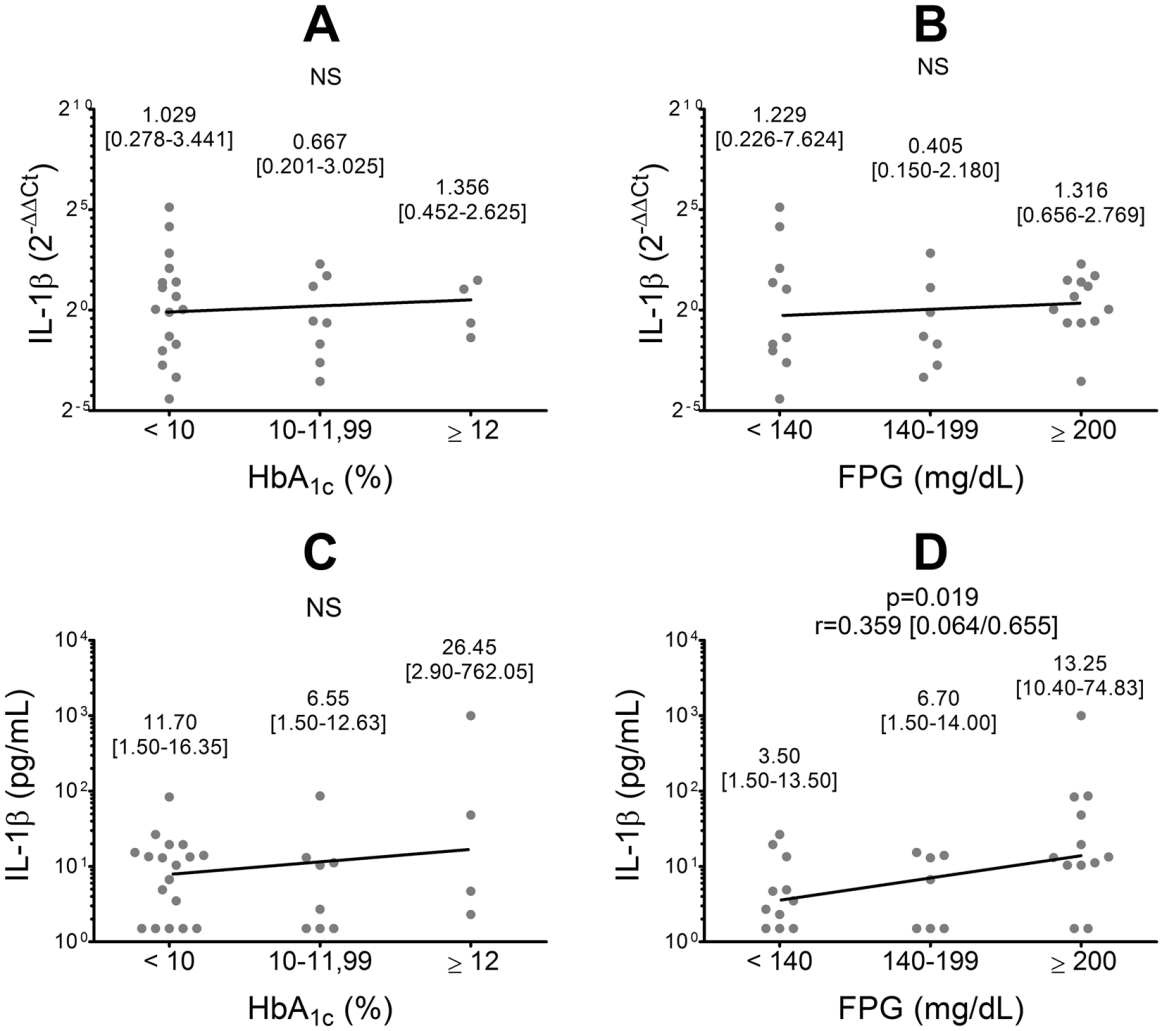
IL-1β mRNA expression (**A,B**) and serum protein levels (**C,D**) at the basal time of the study (pre-intervention group), according to HbA_1c_ and fasting plasma glucose levels. For each data set the median and interquartile range are informed. Statistical evaluation: Multiple linear regression. Covariates: age, gender and weight. HbA_1c_: glycated hemoglobin; FPG: fasting plasma glucose; r: regression coefficient; NS: not significant.

The median IL-1β serum concentration at the beginning of the study was 10.40 pg/mL (interquartile range [IQR]: 1.50–16.35 pg/mL). Thirteen individuals (43.33%) showed values within the reference range (<5 pg/mL) and 17 individuals (56.67%) showed values above it (≥5 pg/mL). At basal time, serum IL-1β levels were positively correlated with FPG (Fig. 1). IL-1β mRNA expression did not correlate with any biochemical-chemical variable analyzed.

**Figure 2:**
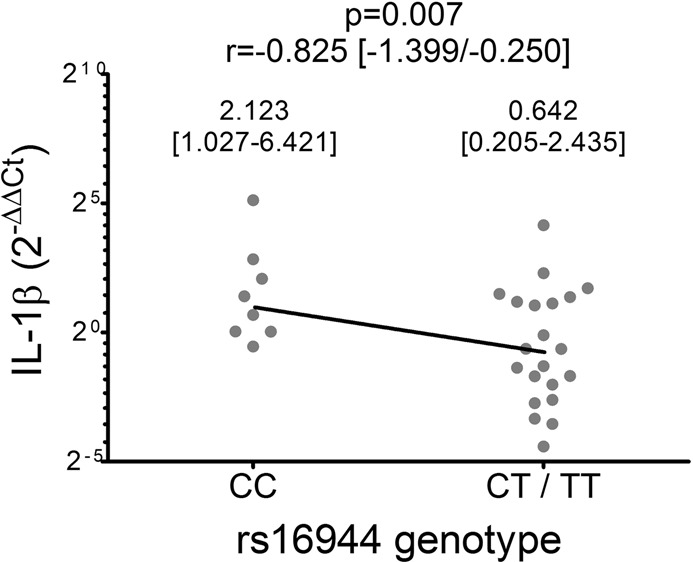
IL-1β mRNA expression at the basal time of the study (pre-intervention group), according to rs16944 genotype. For each data set the median and interquartile range are informed. Statistical evaluation: Multiple linear regression. Covariates: age, gender and weight. r: regression coefficient.

The genotypic frequencies of rs16944 in the IL-1β gene were as follows: 8 individuals were CC (26.7%), 15 individuals CT (50.0%) and 7 individuals TT (23.3%). The SNP was in good accordance with the expected genotype distributions calculated by the Hardy–Weinberg law (p = 0.995). The study through a dominant model showed a significant association between the presence of the T polymorphic allele (genotypes CT and TT) and lower IL-1β mRNA expression (Fig. 2).

### Post-intervention group analysis

The prospective study was carried out in the 27 individuals who finished the protocol. This population consisted of 7 women (25.90%) and 20 men (74.10%), with a mean age of 49.48 years (SD = 12.33; range: 23-69 years). Table 1 shows the biochemical and clinical profile of the post-intervention group. The individuals reached the target of glycemic control after 6 months of treatment and maintained it after 12 months, as demonstrated by the significant decrease in FPG and HbA_1c_ at post-intervention stages. When other biochemical-clinical characteristics were analyzed, a significant decrease was observed in weight and BMI after 6 months of treatment, followed by a significant increase in HDL-C after 12 months of treatment. There were no significant changes in any of the other metabolic variables.

**Figure 3:**
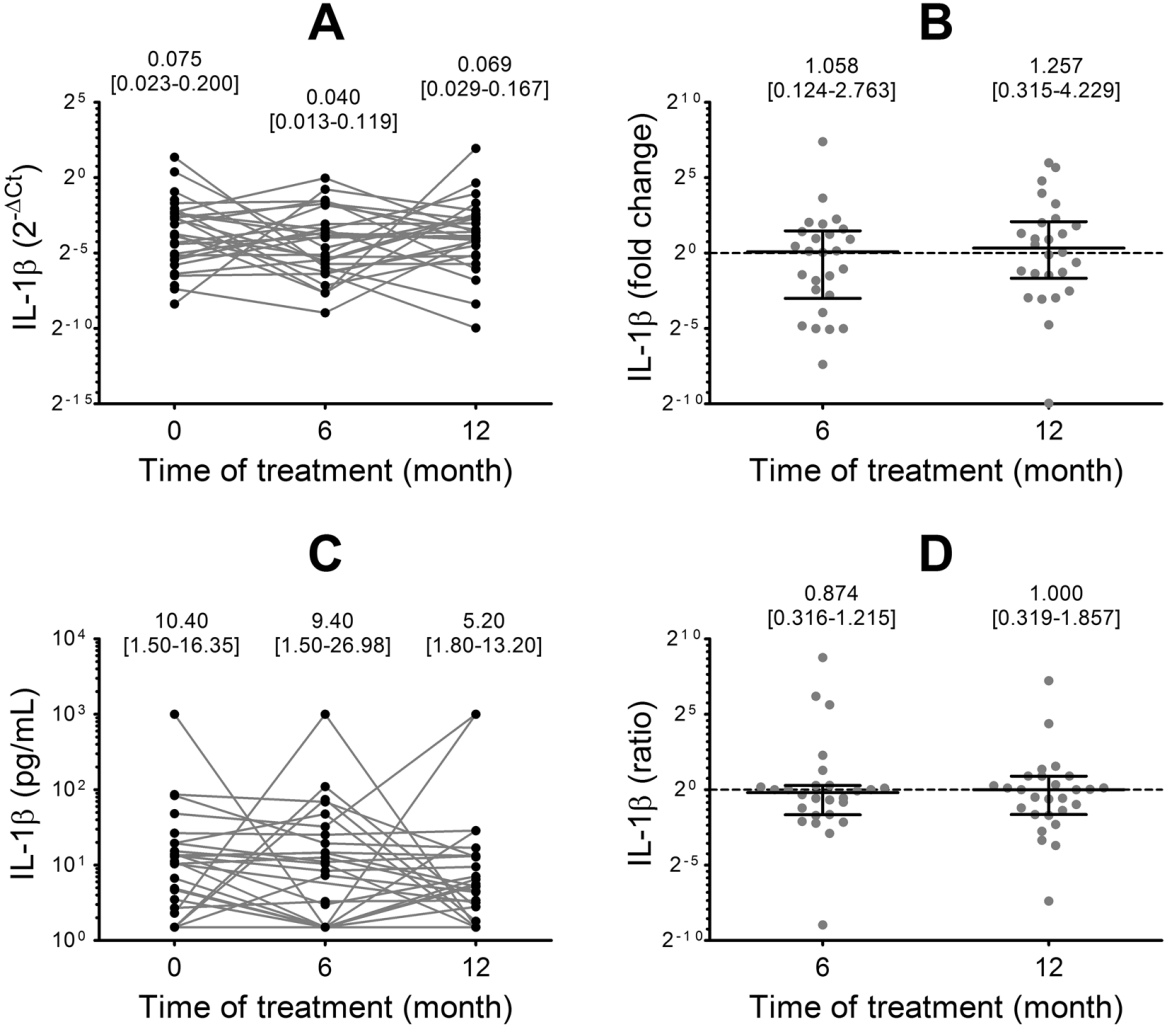
IL-1β mRNA expression and serum levels at each time of the study and variation after 6 and 12 months of treatment. (**A**) Levels of IL-1β mRNA expression and (**B**) fold change after treatment. (**C**) Levels of IL-1β serum protein and (**D**) ratio of variation after treatment calculated as the serum protein level after treatment (at 6 and 12 months) divided by the initial serum protein level (0 months). For each data set the median and interquartile range are informed. Statistical evaluation: Friedman test. All comparisons were not significant.

**Figure 4:**
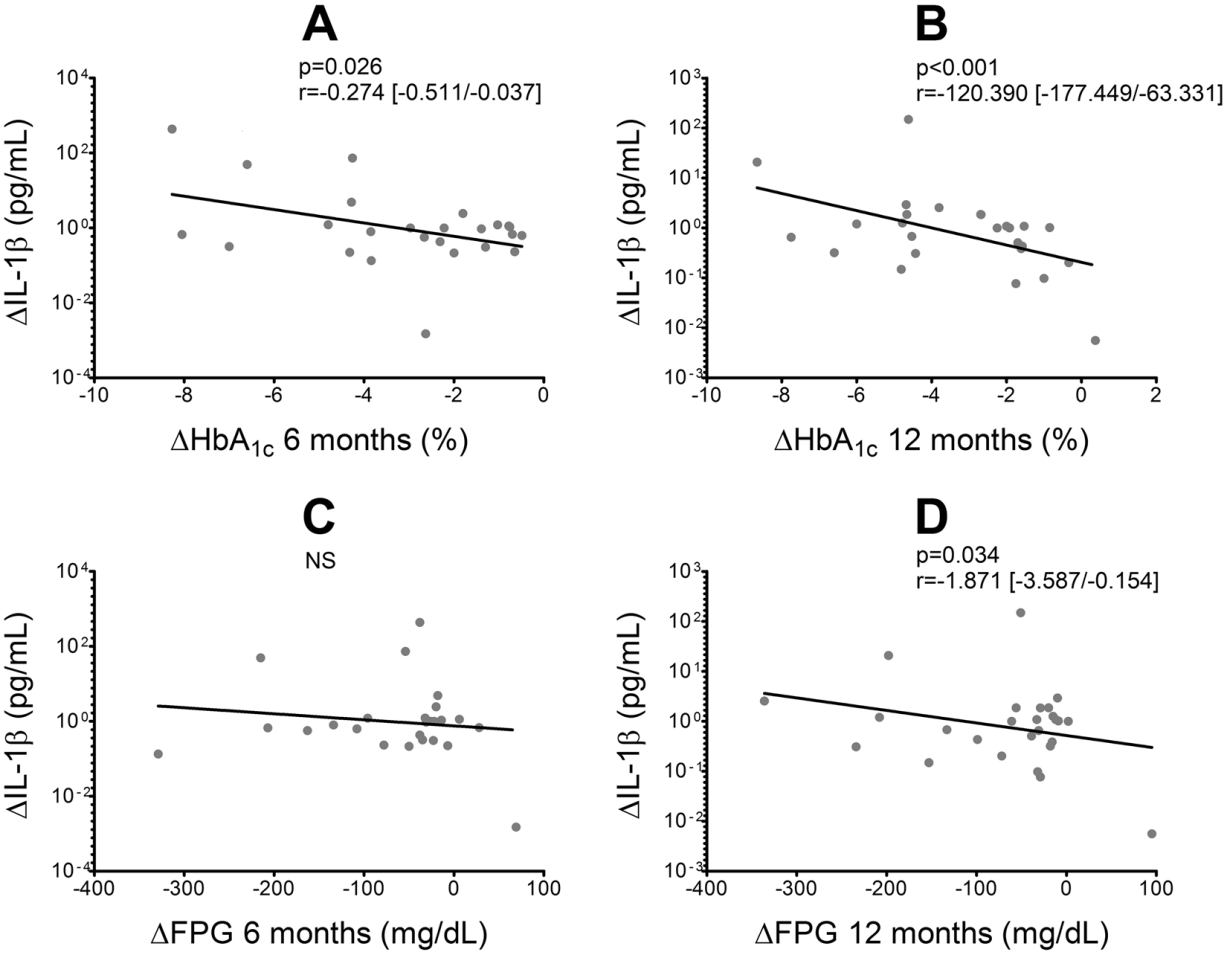
Variations in IL-1β serum levels in function of the variations in HbA_1c_ (**A,B**) and fasting plasma glucose (**C,D**) levels after 6 and 12 months of treatment. Statistical evaluation: Multiple linear regression. For graphic purposes, the variations in IL-1β serum levels are shown as the differences of the logarithms. Covariates: age, gender and weight. HbA_1c_: glycated hemoglobin; FPG: fasting plasma glucose; r: regression coefficient; NS: not significant.

Of note, even if IL-1β mRNA expression and serum protein levels presented no significant differences with respect to pre-intervention time (Fig. 3), the sharper decreases in FPG and HbA_1c_ after treatment were associated with greater reductions in IL-1β protein levels (Fig. 4).

## Discussion

The inflammatory response could be a critical pathophysiological component in the development of T2D^19^. Among inflammatory cytokines, IL-1β is considered the key pro-inflammatory factor in T2D-associated inflammation, as high glucose levels induce metabolic stress and an inflammatory response involving cytokine secretion, islet immune-cell infiltration and β-cell apoptosis^20^, particularly by overproduction of IL-1β^21^.

Several preclinical and clinical studies have shown the usefulness of IL-1 antagonist therapy^9,19,22,23^. For instance, trials have shown that blocking IL-1 signaling by recombinant IL-1 receptor antagonist Anakinra^22^ or neutralizing IL-1β through monoclonal antibodies Canakinumab^24^, Gevokizumab^25^ and LY2189102^26^ result in an improvement in glycemic control in T2D patients^27^. However, it was recently reported that Canakinumab failed to prevent progression from prediabetes to T2D^28^.

In the present work we assessed mRNA expression and serum protein levels of IL-1β in newly diagnosed T2D individuals with hyperglycemia and after 6 and 12 months of treatment, when they achieved their glycemic target control. Also, we evaluated the association between the rs16944 genotype with mRNA expression, serum levels of IL-1β, biochemical and clinical variables.

IL-1β is produced mainly by monocytes (MO), and hyperglycemia may regulate its production through the assembly of a nucleotide-binding domain and leucine-rich repeat containing family pyrin (NLRP3) of the inflammasome, a cytosolic multi-protein platform where the inactive pro-IL-1β is cleaved into an active form via caspase-1 activity^29,30^. Recently, Kousathana *et al*.^31^ provide evidence for a qualitative impairment of cytokine production and innate immune response in T2D individuals. They observed defective production of IL-1β by circulating monocytes in the hyperglycemic state, due to decreased activation of the NLRP3 inflammasome, which were restored by a proper glycemic control. The referred study highlighted the importance of glucose management in the maintenance of appropriate immune function in patients with T2D^31^. In addition, Misaki *et al*. studied healthy and preclinical middle-aged non-overweight and overweight Japanese men, and found that plasma IL-1β concentrations were significantly associated with glucose levels up to 125 mg/dL^13^. Worth highlighting, the current study has replicated those results in a T2D glycemic normalization follow-up study for the first time. We studied a cohort of hyperglycemic states (≥126 mg/dL) and observed that individuals with higher FPG levels presented higher levels of serum IL-1β in the pre-intervention group, in agreement with the above-mentioned findings by Kousathana *et al*.^31^. Also, Ruscitti *et al*. observed a significant increase in NLRP3 expression and IL-1β production by MO of patients affected by T2D after 24 h of incubation with different concentrations of glucose^32^. In other words, serum pro-inflammatory cytokines, including IL-1β, were significantly higher in individuals with hyperglycemia compared with controls, associated to significantly increased oxidative stress^33^.

It should be considered that IL-1β expression regulation is highly complex and depends on at least two independent mechanisms, one at the level of transcriptional activation and another one at the level of translational efficiency^34^, which could justify the dissociation between mRNA and protein levels reported here. In contrast, the association between glucose levels and serum protein levels of IL-1β suggests that glucose levels are one of the factors exerting an effect on the expression of IL-1β at the level of the translation.

In addition, we found that after treatment, a significant decrease was observed in FPG and HbA_1c_, with no significant changes in IL-1β serum levels. However, an association was found between the greater decreases in FPG and HbA_1c_ and the greater –though non-significant– increases in the circulating levels of IL-1β observed after treatment. The same finding was reported by Kousathana *et al*., who provide evidence for the restored production of IL-1β by circulating monocytes after proper glycemic control probably due to activation of the NLRP3 inflammasome^31^. As an alternative explanation for our findings, endoplasmic reticulum (ER) dysfunction in pancreatic β cells is one of the pathogenic mechanisms which determine the development of T2D and is prompted by hyperglycemia, among other mechanisms^35,36^. Considering that hyperglycemia may have the same effects on ER and circulating monocytes, the decrease in glucotoxicity may favor the production of proteins such as IL-1β. This could justify the negative association between the variations in IL-1β serum levels and the variations in FPG and HbA_1c_ after treatment, not related to changes in IL-1β mRNA expression.

Our current results also show that the rs16944 genotype (-511C/T) in the promoter of the IL-1β gene may have an effect on gene transcription, where the presence of the polymorphic T allele was associated with a lower expression of mRNA. This is in agreement with previous publications were a specific IL-1β haplotype (-3893G, -1464G, -511C and -31T) in the promoter region of IL-1β was associated with increased IL-1β mRNA^37^. Moreover, significantly lower secretion of IL-1β was found to be associated with CTC haplotype -1470G/C, -511T/C, and -31C/T in the promoter of the gene^38^. Polymorphisms in the IL-1β gene promoter region alter lipopolysaccharide (LPS) effects on IL-1β gene transcription, leading to susceptibility to inflammatory diseases^39^. Wen *et al*. have suggested that IL-1β promoter haplotypes influence the expression and transcriptional activity of the IL-1β gene after LPS exposure and observed gene upregulation in subjects with haplotype GCT (-1470G, -511C, and -31T)^40^.

It must be taken into consideration that this study was carried out in a population with different proportions of men and women and further and longer studies should be undertaken to determine the effect of ER stress on IL-1β production. Also, longer after-treatment studies may prove interesting to determine the effect of hyperglycemia normalization on the expression of IL-1β.

To resume, this report shows IL-1β mRNA expression level association with the genotype of the rs16944 SNP present in the promoter of the IL-1β gene, a positive association between IL-1β protein levels and FPG and a negative association between variations in IL-1β protein levels and variations in FPG and HbA_1c_.

## Conclusions

To our knowledge, this is the first follow-up study evaluating IL-1β mRNA expression and serum levels in hyperglycemic T2D individuals and after glycemic normalization treatment. Even if longer after-treatment studies may prove interesting to determine the effect of hyperglycemia normalization on the expression of IL-1β, the current results contribute to the knowledge of the relationship between inflammation and changes in glucose metabolism in T2D. Individuals with T2D constitute a heterogeneous group, for which treatment objectives must be individualized. In this context, it is crucial to find new molecular targets for the development of new drugs, guiding the therapeutic strategy in a personalized way (pharmacogenetics).

## Disclosure

The authors declare that there is no conflict of interest that could be perceived as prejudicing the impartiality of the research reported.

The study was not pre-registered in a national or international database.

## Supplementary information

Supplementary Information.

## Data Availability

The data that support the findings of this study are available from the corresponding author upon request. Not public available due to privacy patient restriction.
