# Supplementary material for: Relationship between the IL-1β serum concentration, mRNA levels and rs16944 genotype in the hyperglycemic normalization of T2D patients
Source: Sci Rep. 2020 Jun 19;10:9985. doi: 10.1038/s41598-020-66751-x (PMC7305205; doi:10.1038/s41598-020-66751-x)
Supplement: Supplementary file 1 — Supplementary Information. [file 41598_2020_66751_MOESM1_ESM.docx]

**RELATIONSHIP BETWEEN THE IL-1Β SERUM CONCENTRATION, mRNA LEVELS AND RS16944 GENOTYPE IN THE HYPERGLYCEMIC NORMALIZATION OF T2D PATIENTS.**

**Andrea Elena Iglesias Molli, María Fernanda Bergonzi, Mónica Spalvieri; María Amelia Linari, Gustavo Daniel Frechtel & Gloria Edith Cerrone**

**REFERENCES**

1. Zimmet, P., Alberti, K.G., Shaw, J. Global and societal implications of the diabetic epidemic. *Nature* **414**:782-7 (2001).

2. Schargrodsky, H., *et al*. CARMELA: assessment of cardiovascular risk in seven Latin American. *Am J Med.* **121**:58-65(2008).

3. De Sereday, M.S., *et al*. Prevalence of diabetes, obesity, hypertension and hyperlipidemia in the central area of Argentina. *Diabetes Metab.* **30**:335-9 (2004).

4. Gallagher, E.J., Le Roith, D. & Karnieli, E. The metabolic syndrome-from insulin resistance to obesity and diabetes. *Endocrinol Metab Clin North Am*. **37:** 559-79 (2008).

5. Banerjee, M. & Saxena, M. Interleukin-1 (IL-1) family of cytokines: role in type 2 diabetes. *Clin Chim Acta* **413**:1163-70 (2012).

6. Sepehri, Z. *et al.* (2017) Inflammasomes and type 2 diabetes: An updated systematic review. *Immunol Lett.* **192**:97-103.

7. Dinarello, C.A. Immunological and inflammatory functions of the interleukin-1 family. *Annu Rev Immunol.* **27**:519-50 (2009).

8. Maedler, K., *et al*. Low concentration of interleukin-1 ß induces FLICE-inhibitory protein-mediated beta cell proliferation in human pancreatic islets. *Diabetes* **55**:2713–22 (2006).

9. Donath, M.Y., *et al*. Islet inflammation in type 2 diabetes: from metabolic stress to therapy. *Diabetes Care* **31**:161-4 (2008).

10. Maedler, K., *et al.* Glucose induced ß cell production of IL-1 ß contributes to glucotoxicity in human pancreatic islets. *J Clin Invest.* **110**:851–60 (2002).

11. Fukaya, N., Mochizuki, K., Shimadam, M. & Godam, T. The α-glucosidase inhibitor miglitol decreases glucose fluctuations and gene expression of inflammatory cytokines induced by hyperglycemia in peripheral leukocytes. *Nutrition* **25**:657-67 (2009).

12. Dasu, M.R., Devaraj, S. & Jialal, I. High glucose induces IL-1β expression in human monocytes: mechanistic insights. *Am J Physiol Endocrinol Metab*. **293**:337-46 (2007).

13. Misaki, Y., *et al.* Plasma interleukin-1β concentrations are closely associated with fasting blood glucose levels in healthy and preclinical middle-aged nonoverweight and overweight Japanese men. *Metabolism* **59**:1465-71 (2010).

14. Kallio, P., *et al.* Inflammation markers are modulated by responses to diets differing in postprandial insulin responses in individuals with the metabolic syndrome. *Am J Clin Nutr.* **87**:1497-1503 (2008).

15. Doria, A., Patti, M.E. & Kahn, C.R. The emerging genetic architecture of type 2 diabetes. *Cell Metab.* **8**:186-200 (2008).

16. Ballak, D.B., Stienstra, R., Tack, C.J., Dinarello, C.A. & van Diepen, J.A. IL-1 family members in the pathogenesis and treatment of metabolic disease: Focus on adipose tissue inflammation and insulin resistance. *Cytokine* **75**:280-90 (2015).

17. Donath, M.Y. & Shoelson, S.E. Type 2 diabetes as an inflammatory disease. *Nat Rev Immunol* **11**:98-107 (2011).

18. Masters, S.L., *et al*. Activation of the NLRP3 inflammasome by islet amyloid polypeptide provides a mechanism for enhanced IL-1beta in type 2 diabetes. *Nat Immunol.* **11**:897-904 (2010).

19. Maedler, K., *et al*. Glucose-induced beta cell production of IL-1beta contributes to glucotoxicity in human pancreatic islets. *J Clin Invest* .**110**: 851-60 (2002).

20. Donath, M.Y., *et al.* Islet inflammation in type 2 diabetes: from metabolic stress to therapy. *Diabetes Care* **31**:161–4 (2008).

21. Larsen, C.M., *et al.* Interleukin-1-receptor antagonist in type 2 diabetes mellitus. *N Engl J Med.* **356**:1517–26 (2007).

22. Ruscitti, P., *et al.* Efficacy of inhibition of IL-1 in patients with rheumatoid arthritis and type 2 diabetes mellitus: two case reports and review of the literature. *J Med Case Rep***. 2**:123 (2015).

23. Dhimolea, E. *Canakinumab mAbs.* **2**:3-13 (2010).

24. Cavelti-Weder, C., *et al.* Effects of gevokizumab on glycemia and inflammatory markers in type 2 diabetes. *Diabetes care* **35**: 1654-62 (2012).

25. Sloan-Lancaster, J., *et al.* Double-blind, randomized study evaluating the glycemic and anti-inflammatory effects of subcutaneous LY2189102, a neutralizing IL-1beta antibody, in patients with type 2 diabetes. *Diabetes care* **36**: 2239-46 (2013).

26. Cavelti-Weder, C., *et al*. Development of an Interleukin-1β Vaccine in Patients with Type 2 Diabetes. *Mol Ther.* **24**:1003-12 (2016).

27. Everett, B.M., et al. Anti-inflammatory therapy with canakinumab for the prevention and management of diabetes. *J Am Coll Cardiol.* **21**:2392-2401(2018).

28. Schroder, K., Zhou, R. & Tschopp, J. The NLRP3 inflammasome: a sensor for metabolic danger? *Science* **327**:296–300 (2010).

29. Menu, P. & Vince, J.E. The NLRP3 inflammasome in health and disease: the good, the bad and the ugly. *Clin Exp Immunol.* **166**:1-15 (2011).

30. Kousathana, F., *et al.* Defective production of interleukin-1 beta in patients with type 2 diabetes mellitus: Restoration by proper glycemic control. *Cytokine* **90**:177-184 (2017).

31. Ruscitti, P., *et al.* Monocytes from patients with rheumatoid arthritis and type 2 diabetes mellitus display an increased production of interleukin (IL)-1β via the nucleotide-binding domain and leucine-rich repeat containing family pyrin 3(NLRP3)-inflammasome activation: a possible implication for therapeutic decision in these patients. *Clin Exp Immunol.* **182**:35-44 (2015).

32. Li, J., Huang, M. & Shen, X. The association of oxidative stress and pro-inflammatory cytokines in diabetic patients with hyperglycemic crisis. [*J Diabetes Complications*](https://www.ncbi.nlm.nih.gov/pubmed/25044235) **28**:662-6 (2014).

33. Schindler, R., Clark, B.D., & Dinarello, C.A. Dissociation between interleukin-1 beta mRNA and protein synthesis in human peripheral blood mononuclear cells. *J Biol Chem* **265**:10232–7 (1990).

34. Hajmrle, C., *et al.* Interleukin-1 signaling contributes to acute islet compensation. *JCI Insight* **1**: e86055 (2016).

35. Dror, E., *et al*. Postprandial macrophage-derived IL-1β stimulates insulin, and both synergistically promote glucose disposal and inflammation. *Nat Immunol.* **18**:283-292 (2017).

36. Eizirik, D.L., Cardozo, A.K. & Cnop, M. The Role for Endoplasmic Reticulum Stress in Diabetes Mellitus. *Endocrine Reviews* **29**:42–61 (2008).

37. Hotamisligil, G.S. Endoplasmic Reticulum Stress and the Inflammatory Basis of Metabolic Disease. *Cell* **140**:900–17 (2010).

38. Sage, A.T., *et al.* Metabolic syndrome and acute hyperglycemia are associated with endoplasmic reticulum stress in human mononuclear cells. *Obesity (Silver Spring)* **20**:748-55 (2012).

39. Landvik, N.E., *et al.* A specific interleukin-1beta haplotype correlates with high levels of IL1B mRNA in the lung and increased risk of non-small cell lung cancer. *Carcinogenesis* **30**:1186–92 (2009).

40. Wen, A.Q., *et al*. Clinical relevance of IL-1beta promoter polymorphisms (-1470, -511, and -31) in patients with major trauma. *Shock* **33**:576-82 (2010).

41. Hall, S.K., *et al.* Correlation of polymorphic variation in the promoter region of the interleukin-1 beta gene with secretion of interleukin-1 beta protein. *Arthritis Rheum* **50**:1976-83 (2004).

42. Wen, A.Q., *et al.* Effects of haplotypes in the interleukin 1beta promoter on lipopolysaccharide-induced interleukin 1beta expression. *Shock* **26**:25–30 (2006).

43. Berthier, F., Lambert, C., Genin, C. & Bienvenu, J. Evaluation of an automated immunoassay method for cytokine measurement using the Immulite Immunoassay system. *Clin Chem Lab Med*. **37**: 593–599 (1999).

44. Schmittgen, T.D. & Livak K.J. Analyzing real-time PCR data by the comparative Ct method. *Nature Protocol.* **3**:1101-8 (2008).

45. Gustincich, S., Manfiolett, G., Del Sal, G., Schneider, C. & Carninci, P. A fast method for high-quality genomic DNA extraction from whole human blood. *Bio Techniques* **11**:298-302 (1991).
